# The effect of increasing availability of vegetarian meals on their sales in worksite cafeterias: a stepped-wedge cluster randomised controlled trial

**DOI:** 10.1186/s12966-026-01889-x

**Published:** 2026-04-15

**Authors:** Elisa Becker, Emma E Garnett, Peter Scarborough, Steven Cummins, Bea Savory, Oliver Huse, Claire Thompson, Jessica Brock, Michael Clark, Jessica Renzella, Martin White, Rachel Pechey

**Affiliations:** 1 Nuffield Department of Primary Care Health Sciences, University of Oxford, Radcliffe Observatory Quarter, Woodstock Road, Oxford, OX2 6GG UK; 2 NIHR Oxford Health Biomedical Research Centre, Oxford, UK; 3 Population Health Innovation Lab, Department of Public Health, London School of Hygiene & Tropical Medicine, Environments & Society, London, UK; 4 School of Health, Medicine and Life Sciences, University of Hertfordshire, Hertfordshire, UK; 5 Smith School of Enterprise and the Environment, School of Geography and Environment, University of Oxford, Oxford, UK; 6 Institute of Metabolic Sciences, MRC Epidemiology Unit, Clinical School, University of Cambridge, Cambridge, UK

## Abstract

**Background:**

Reducing meat consumption is critical for planetary health and could benefit public health. Restructuring food environments – such as increasing vegetarian meal availability – has been shown to influence dietary choices in online and university canteen settings. However, evidence from more diverse populations is limited.

**Methods:**

We conducted a stepped-wedge cluster-randomised controlled trial in six English worksite cafeterias over seven weeks. The intervention involved replacing one meat-based meal on the lunch menu with a vegetarian option. Likelihood of selecting a vegetarian meal (primary outcome) was analysed in a logistic regression with random intercept for cafeteria and fixed effect for time. Secondary outcomes included nutritional and sustainability markers (e.g., kilocalories, greenhouse gas (GHG) emissions), revenue, and food waste. Interviews with cafeteria staff and customers explored intervention acceptability and barriers.

**Results:**

The intervention encompassed 26,170 meal sales over 42 site-weeks. The intervention increased the likelihood of selecting a vegetarian meal by 41% [95% CI: 28 to 55]. Per-meal kilocalories were reduced by 26.1 [95% CI -34.4 to -17.7], and per-meal GHGs by 0.16 kg CO_2_-eq [-0.22 to -0.11]. No significant negative impacts on business outcomes such as cafeteria revenue (-98.43 GBP [-436.03 to 239.17]) or food waste (-8.25 kg [-48.38 to 31.87]; -61.64 GBP [-306.80 to 183.53]) were found. Customers and staff reported that the intervention was acceptable, non-intrusive, and easy to implement, with concerns about customer dissatisfaction and food waste not confirmed by our data. Suggestions for improvement included greater attention to pricing and taste to further encourage uptake.

**Conclusions:**

Increasing the availability of vegetarian meals in cafeterias can significantly shift food choices and reduce environmental impact and calories consumed, without compromising business outcomes, and should be considered an effective strategy for sustainability and public health in diverse food service settings.

**Trial registration number:**

This study is registered with the ISRCTN registry, ISRCTN36918695.

**Supplementary Information:**

The online version contains supplementary material available at 10.1186/s12966-026-01889-x.

## Introduction

In more affluent parts of the world meat is overconsumed [1] to the detriment of population and planetary health. Livestock farming is a leading driver of climate change [2–4]. In the UK, in comparison to high meat diets (> 100 g/d), low meat diets are associated with 47% less greenhouse gas (GHG) emissions, 56% less land use, 20% less water use, 43% less eutrophication (a form of nutrient pollution in water bodies [5]) and 31% less biodiversity impact [6]. Additionally, red and processed meat consumption is linked to various adverse health outcomes, including colorectal, pancreatic, and prostate cancers [7] and heart disease [8–10]. Together, these adverse outcomes demand urgent action to shift diets to include more plants and less meat [11].

Food choices, including meat consumption, are often habitual and driven by external cues rather than consumers’ intentions [12, 13]. Therefore, implementing interventions in micro-environments like restaurants or supermarkets (‘nudging’) can influence selections to improve diet-related health and/or sustainability [14, 15]. One such option is altering the availability of a particular set of food options (vegetarian meals in this case), with three possible mechanisms of action [16]: preference, social norms, and attention. First, if more vegetarian meals become available, this increases the likelihood that, for any given customer, the preferred option in the choice set is vegetarian [17]. Second, increased vegetarian meal availability can convey a social norm of vegetarianism amongst customers of that food outlet [18]. Third, although product positioning is not directly targeted in availability interventions, increased numbers of vegetarian options make it more likely that a vegetarian option is placed in a more prominent position within the cafeteria spread, where the option may receive more attention and be selected more often [19, 20]. A fourth, indirect mechanism may involve price: if vegetarian meals are generally cheaper, increasing their number may raise the availability of low-cost options, encouraging price-sensitive customers to choose them.

Availability interventions appear effective in reducing meat consumption across a range of out-of-home settings. A recent supermarket study found that a multi-component intervention – combining increased availability, visibility, and affordability of plant-based products – led to substantial increases in plant-based sales [21]. However, its implementation during Veganuary raises questions about generalisability to less motivated populations. Similarly, availability interventions have shown good acceptability and effectiveness in student canteens [22, 23], increasing vegetarian purchases without reducing overall sales or customer satisfaction. One study also reported reduced greenhouse gas emissions, although with a decrease in nutritional quality [22]. In workplace cafeterias, findings have been more mixed: one study reported reduced meat sales, while another showed limited effects, potentially due to implementation challenges [24]. Together, these findings highlight the need for controlled trials in workplace cafeterias that assess not only effects on vegetarian meal sales, but also on nutritional and environmental outcomes, business metrics, and the perspectives of both staff and customers.

To gain a broad understanding of the effects of availability interventions, this study evaluated the impact of an intervention in which worksite cafeterias increased the relative availability of vegetarian main meals on relative sales of vegetarian meals at lunchtime in a stepped-wedge randomised controlled trial (RCT). We also estimated the effect on meal energy and four different environmental impact markers. We measured business outcomes on revenue, total sales, and food waste and conducted qualitative research on cafeteria customer and staff perceptions of the intervention’s feasibility and acceptability.

## Methods

### Trial design

**Fig. 1:**
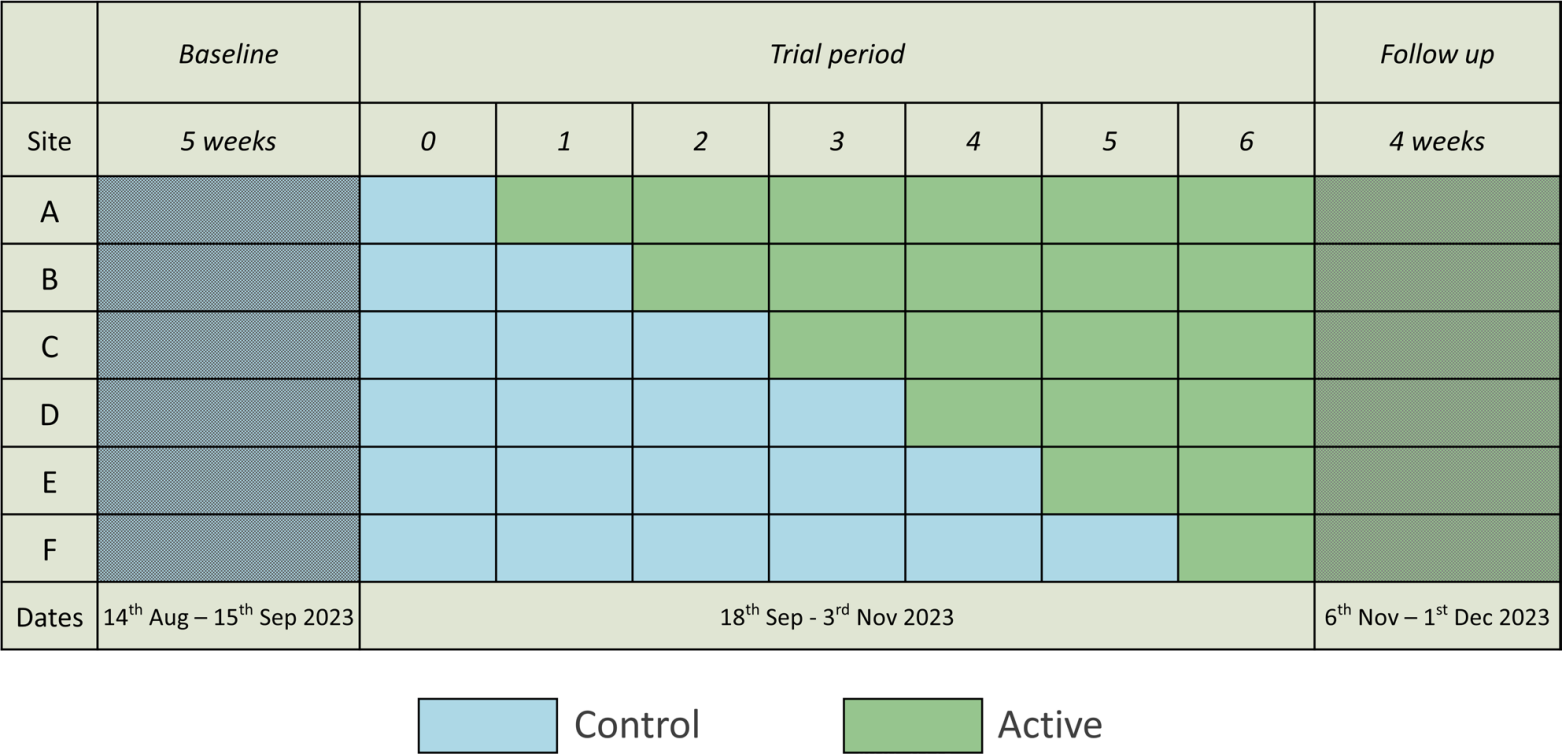
Stepped-wedge intervention schedule

A stepped-wedge RCT was implemented in six worksite cafeterias (clusters) served by one UK catering company, over a period of seven weeks (18th Sep – 3rd Nov 2023), with participating sites randomly assigned to start the intervention in one of six different weeks (see Fig. 1).

Six cafeteria sites (A-F) increased their vegetarian meal availability at different, randomised starting points between week one and six. Prior to the main trial period, data was collected for five weeks of baseline monitoring without the intervention in place. After the main trial period, all sites retained the intervention for a four-week follow-on period, ending on the Dec 1, 2023. This time frame was chosen to avoid the month of December during which food consumption patterns are often more indulgent [25, 26].

### Eligibility criteria and recruitment

To be eligible, cafeterias had to:

- offer at least three main meal options at lunchtime.
- offer at least two meat-based meals and at least one vegetarian meal (to ensure that a choice between meat/fish-based and vegetarian meals was possible at baseline and during the intervention).

If meeting the eligibility criteria varied systematically across the week for any site (e.g., only two meal options every Monday, or only meat- or fish-based meals every Friday), then a site was included if eligibility criteria were met for at least three out of five weekdays. In such cases, the non-eligible day(s) were excluded from calculating weekly sales averages during data analysis. If sites operated on weekends, the intervention was only implemented during weekdays, and weekend data was not included in analyses.

Recruitment took place between April 11 and September 18, 2023, in three stages: eligibility screening, invitation of eligible sites, and pre-trial menu monitoring. Eligibility screening was facilitated by the catering company’s central staff who identified 60 sites based on our eligibility criteria. Invitations to take part in the study were sent out to all 60 sites deemed eligible. Invitation efforts included an initial email sent out by the catering company’s central staff, an online group meeting with interested cafeteria managers at which trial requirements were explained, and 1:1 phone calls and emails to cafeteria managers who had not responded to the initial invitation email. Sites that responded positively to the invitation entered a pre-intervention monitoring period during which they were asked to send photos of weekly menus. These were used to confirm eligibility and test whether sites would be able to comply with the ongoing monitoring requirements of the study (i.e. sending weekly menus).

### Intervention

The intervention was set in six workplace cafeterias across England, located at least 17.1 miles apart. Cafeterias served a range of employee populations, including both manual (e.g., assembly line) and office-based workers across three different companies in two sectors (car manufacturing and logistics). Demographic data on the workforce at different sites were not available. Cafeteria customers were not informed of the intervention.

The intervention was delivered by cafeteria managers who were asked to exchange one of their meat-based meals (including fish) from their lunch main meal offer for a vegetarian meal each day during active intervention weeks, thereby increasing vegetarian and reducing meat-based meal availability while keeping the total number of lunch main meals constant. Cafeteria managers received no training to be able to deliver the intervention and were not given instructions on the type or quantity of vegetarian or meat-based dishes to prepare.

The intervention focused on lunch main meals prepared on-site, and did not alter any other items on sale in the cafeterias. Meal prices were not altered and varied across sites according to their own business models.

EB conducted fidelity checks by reviewing photographs of all cafeteria menus (starting at least six weeks prior to trial start date and supported by a small shopping voucher pay-out to cafeteria managers for each menu sent), and one announced visit per site (completed by either EB, or EG). Weekly telephone calls by EB supported cafeteria managers with menu planning and checked for dish sell-outs. Sell-outs were rare, occurred only late in shifts, and were not accounted for in analyses.

Sites maintained the intervention until 1 Dec 2023, creating a non-randomised four-week intervention period following the main trial period (Fig. 1). This was excluded from the primary analysis but included in one sensitivity analysis.

### Outcomes

The primary outcome was the proportion of vegetarian out of total meals sold. Secondary outcomes were total meals sold per week, energy (kcal), fat (g), saturated fat (g), sugar (g), salt (g), protein (g), fibre (g), carbohydrates (g), greenhouse gas emissions (kg CO2-eq), biodiversity loss (species.year*10^− 14^), eutrophication potential (gPO4-eq), water scarcity (L-eq), and price (GBP) per meal sold, portion size (g), cafeteria weekly revenue (GBP) from meals. We could not assess effects on profit margins due to a lack of data on ingredient costs. We also analysed food waste (both in kg and GBP per week). Finally, we analysed proportion of vegetarian non-main meal items (e.g., soups, snacks) sold at lunchtime, as well as total sales of these items to assess potential spill-over effects of meat purchases to other foods offered.

Most outcome measures were derived from sales data, with the following exceptions: meal energy content was separately provided by the catering company for each meal and was routinely estimated as per calorie labelling regulations in the out of home sector for England [27]. Environmental impact of meals was calculated by linking ingredients for each meal to agri-environmental databases containing information on the environmental impacts of producing different agricultural commodities (following methods from Clark et al. [28]). Because ingredient sourcing details (e.g. on origin or production method) were not available, these calculations used global average environmental impacts for each ingredient. The ingredients lists were provided by the catering company. Food waste was analysed from plate and kitchen waste records that were routinely collected by site managers (supplied in total kg and total GBP waste per site per week). We conducted three sensitivity analyses:

Per-protocol analysis: Using weekly menu photographs, we identified 68 site-days where vegetarian meal availability was increased as intended (i.e., ‘per protocol’) out of 83 with an intention to treat. After excluding 15 days on which vegetarian meal availability was not increased as per intervention instructions, four site-weeks were left with insufficient data (< 3 days), leaving 38 site-weeks (out of 42) for the per-protocol analysis.

Secular time trend analysis: To assess potential time trends in vegetarian sales, we analysed centrally provided sales data from non-participating sites matched by sales volume to each intervention site. Each matched site was assigned the same intervention start week as its pair, and the primary mixed-effects model was applied to their sales data. This was a ‘negative control’ sensitivity analysis – we hypothesised that the model would find no effect of the intervention (since there was no intervention in this set of cafeterias). Any effect size would be due to residual confounding from time trends in vegetarian sales, which could also affect the primary analysis.

Extended time period analysis: To explore intervention persistence and increase statistical power, we applied the primary model to an expanded dataset covering five pre-trial baseline weeks (no intervention), the seven-week trial period, and four post-trial weeks (all sites under intervention). The additional weeks were not subject to randomisation.

### Randomisation and masking

A computer-generated random number sequence (www.randomizer.org) was used by EB to generate the order in which participating sites started implementing the intervention. EB then informed each cafeteria site of their respective start date. Because customers were not informed of the intervention, no further masking was applied.

### Process evaluation

To assess acceptability and feasibility of the intervention, cafeteria and customer staff were intercepted at the cafeteria sites after the trial period (February to May 2024) to avoid influencing customer choice during the trial and invited to in-person or telephone-based interviews. Process evaluation interviews were carried out with *n* = 14 intervention recipients (i.e., cafeteria customers) and *n* = 3 implementers (i.e., cafeteria staff) in three cafeterias (selected to include both manufacturing and office-based settings). Interviews lasted an average of 24 min.

Participants provided informed consent prior to participation. Separate topic guides were developed for recipients and implementers, designed in consultation with public involvement groups and refined iteratively during data collection. Grounded in realist principles, questions addressed experiences with the intervention, perceived impact and effectiveness, and potential scalability and optimisation. Ethical approval for this part of the study was obtained from the London School of Hygiene and Tropical Medicine Research Ethics Committee (Ref: 29553).

Audio-recordings were transcribed verbatim. Fieldnotes and photographs of the cafeterias aided data analysis. Data were coded and analysed in NVivo 14 using a General Inductive Approach [29]. To ensure validity of the emerging coding framework, a subset of the data was double-coded, and the framework itself was developed iteratively in consultation with the broader research team. Analysis focused on intervention acceptability, implementation barriers and optimisation.

### Statistical analysis

The effect of the intervention on all outcome variables was evaluated using mixed-effects models (binomial for binary outcomes, linear otherwise) with random intercepts for sites and controlling for a linear fixed effect of week.

Because the number of sites that were eligible and had the operational capacity to take part was relatively low, we took a pragmatic approach to power calculation, calculating achieved power from the biggest sample we could possibly obtain. This power calculation was pre-registered in the analysis plan which can be found under DOI 10.17605/OSF.IO/DSFGZ.

Statistical analyses were carried out in RStudio, version 2024.12.1.

### Deviations from pre-registered analysis plan

As pre-registered, we used consistent model structures across outcomes, with minor adjustments: we used linear models for continuous outcomes, and transaction-level data (rather than weekly averages) to maximise granularity. Pre-registered outcomes like ‘weekly energy sold’ were thus analysed as ‘per-meal energy’.

Pre-registered analyses on environmental impact and energy of non-meal purchases were not possible due to missing recipe data for these items. Instead, we analysed effects on vegetarian sales proportion and number of non-meal items sold at lunch.

We also added an unregistered analysis of meal price to complement revenue outcomes and assess potential effects on consumers.

To assess potential bias from changes in portion size, we additionally analysed portion size and re-ran nutritional and environmental outcomes per 100 g. Finally, we analysed additional per-meal nutritional outcomes (fat, saturated fat, sugar, salt, protein, fibre, and carbohydrates), which were not available at the time of pre-registration.

## Results

**Fig. 2:**
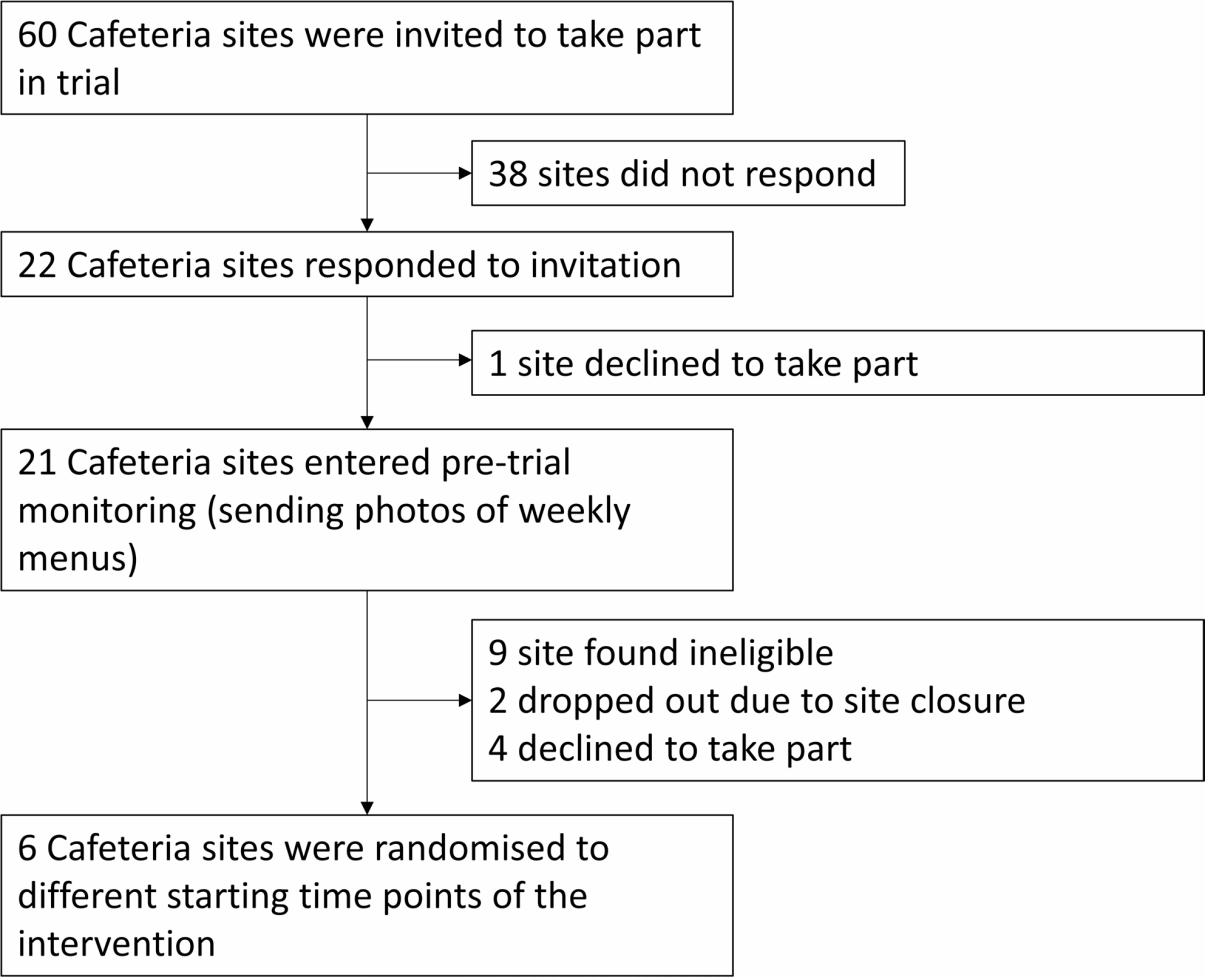
Site recruitment flow diagram

Of the 60 sites identified as eligible and invited to take part, 22 sites responded, and 21 agreed to take part in pre-trial monitoring. During monitoring, ten sites were found to be ineligible based on menu content, two sites withdrew due to imminent closure, and four declined further participation (Fig. 2). The remaining six eligible sites were randomised to staggered intervention start dates (Fig. 1).

All six cafeterias completed the study. The data for analysis comprised 26,170 meals sold, grouped into 42 site-weeks. Across all sites, fidelity checks identified 15 days on which the intervention had not been implemented to protocol, out of 78 days on which sites would have been both eligible and given an instruction to implement the intervention (81% fidelity). These days are included in our primary analysis but removed from the per-protocol analysis.

Participating cafeterias served a mix of office and manufacturing work sites and varied in size and sales volume (70 to 1800 meals per week) and baseline vegetarian meal sales proportion (7% to 27%). Full site characteristics can be viewed in Table 1.

**Table 1:** Site characteristics of participating worksite cafeterias at baseline (five weeks prior to trial start)

Site characteristics at baseline							
Site	Work Type	Weeks In Inter-vention	Non-eligible Days	Meals Sold/Week	Vegetarian Sales Ratio	Total meal options /day	Meal price (GBP)
				Mean (SD)	Mean (SD)	Mean (SD)	Mean (SD)
A	Office	6	14	730.8 (108)	0.25 (0.07)	5.9 (1.3)	3.34 (1.21)
B	Manufacturing	5	3	61 (20)	0.11 (0.05)	3.8 (1.6)	3.19 (0.39)
C	Both	4	7	194.2 (24.3)	0.29 (0.07)	3.7 (0.8)	2.91 (1.16)
D	Both	3	2	472.2 (82.9)	0.22 (0.14)	7.2 (1.7)	2.71 (1.02)
E	Manufacturing	2	1	322.4 (29.7)	0.22 (0.03)	4.2 (1.1)	3.21 (0.79)
F	Both	1	0	1564.6 (206.9)	0.31 (0.03)	6 (0.2)	2.71 (0.8)

**Fig. 3:**
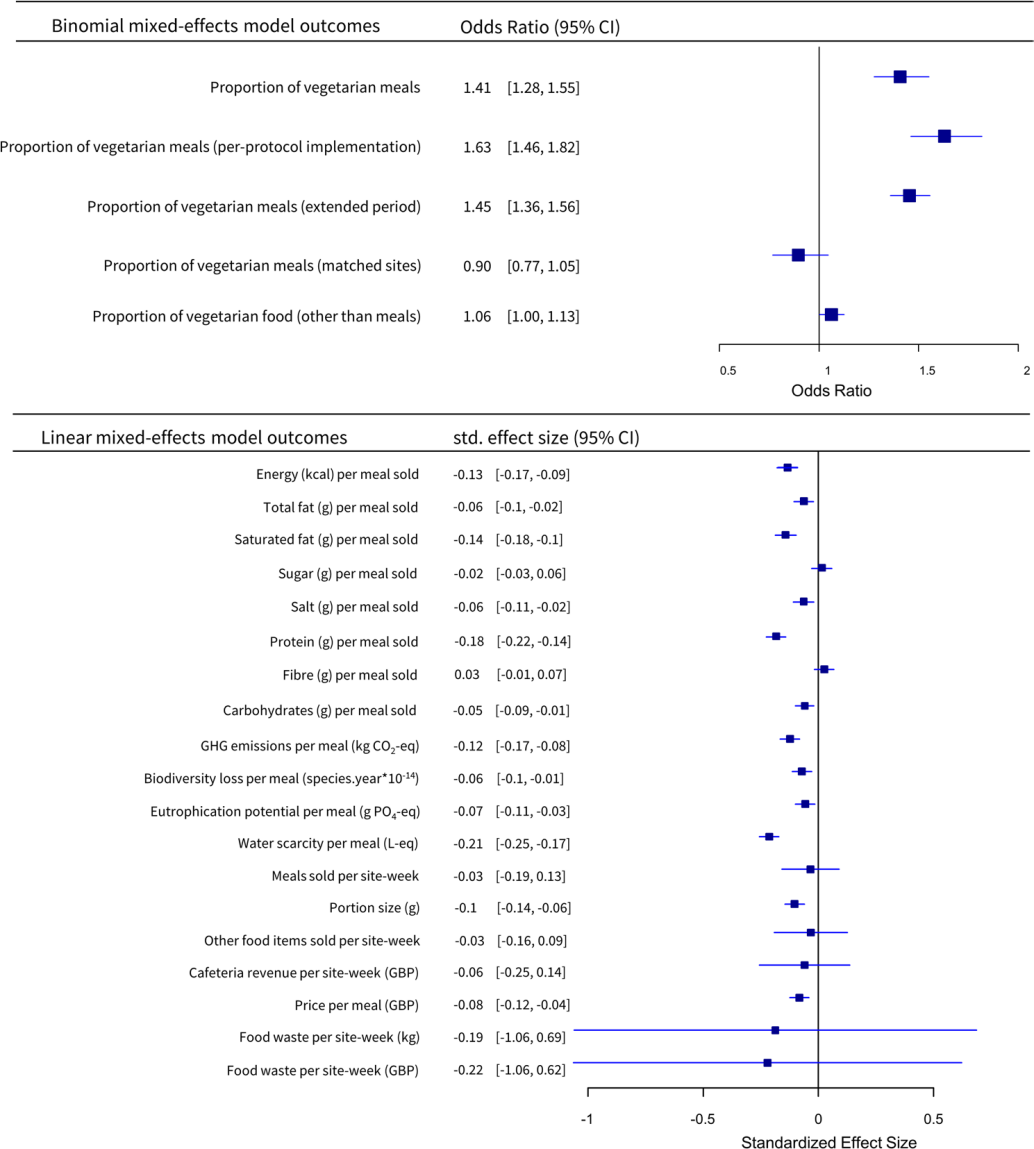
Forest plot of standardised estimates of all outcomes. Note: analyses of price, fat, saturated fat, sugar, salt, protein, fibre, carbohydrates per meal, and portion size were not pre-registered

The odds of purchasing a vegetarian meal increased by 41% (95% confidence interval (CI): 28% to 55%) following the intervention (Fig. 3).

Sensitivity analyses confirmed the robustness of the main effect. When limited to confirmed intervention days (per-protocol analysis), the odds for vegetarian meal selection increased 63% (95% CI: 46%–82%). In matched non-participating sites (secular time trend analysis), the odds of selecting vegetarian meals reduced by 10% (-23%– +5%), indicating no comparable trend. Including non-randomised post-trial weeks (extended time period analysis) produced a similar increase in the odds of selecting a vegetarian main.(45%, 95% CI = 36%–56%), with narrower confidence intervals (Fig. 3).

The intervention reduced per-meal kilocalories by 26.1 kcal (-34.4 to -17.7), fat by 0.59 g (-1.00 to -0.18), saturated fats by 0.49 g (-0.64 to -0.34), salt by 0.05 g (-0.08 to -0.02), protein by 2.04 g (-2.52 to -1.57), and carbohydrates by 1.48 g (-2.73 to -0.23). Fibre and sugar content of meals did not significantly change following the intervention. All nutritional outcome analysis with the exception of per-meal kilocalories were not pre-registered.

Each of our four environmental impact metrics were also reduced: per portion GHG reduced by 0.16 kg CO_2_-eq (-0.22 to -0.11), biodiversity loss by 1.49 species.year*10^− 14^ (-2.62 to -0.37), eutrophication potential by 0.71 gPO_4_-eq (-1.14 to -0.28), and water scarcity by 637.4 L-eq (-764.5 to -510.4) during intervention weeks.

To assess potential bias from changes in portion size, we conducted a (not pre-registered) analysis of portion size and found portions were smaller following the intervention (− 13.65 g, 95% CI − 19.49 to − 7.80). Nutritional and environmental outcomes were therefore re-analysed per 100 g; effect sizes were attenuated but directions and statistical significance were unchanged, except for fibre, which was higher per 100 g post-intervention. Full results are reported in the supplementary material.

A £0.08 (-0.12 to -0.04) per-meal price reduction (post-hoc unregistered analysis) was observed, but no other business metrics (weekly revenue, weekly number of meals sold, or food waste) were significantly affected (Fig. 3). The model investigating food waste in GBP resulted in a singular fit, indicating that the random effect for site did not explain additional variance in the outcome. Despite this, the model structure was retained to maintain consistency with the pre-registered analysis plan.

The forest plot in Fig. 3 shows odds ratios (where the outcome is vegetarian sales proportion) and standardised estimates (for all linear models) and 95% CIs for all outcomes. Full model outputs can be found in the supplementary material.

### Process evaluation

From interviews with intervention recipients and implementers three main themes were identified: intervention acceptability, implementation barriers, and suggestions for optimisation.

Interview findings highlighted that both cafeteria staff and customers generally viewed the intervention as acceptable. Customers appreciated the non-intrusive nature of the changes, and staff overall reported that implementation was straightforward within the existing menu structure. Perceptions of acceptability varied by age, with customers believing that younger and office-based employees would be more open to selecting vegetarian meals than older or manual labour staff.

Barriers to the intervention focused on customers’ perceived lack of protein in vegetarian options, tradition and habits, especially among older male customers, and lunch breaks being too short in some cafeterias. Staff in some sites raised concerns about vegetarian dishes leading to higher workload and food waste (although at other sites staff said workload had not increased).

To improve the intervention, customers suggested that to encourage selection of vegetarian options, taste and price should be improved, and information on the environmental benefit and suggestions by friendly staff would benefit decision making. Table 2 presents the main themes and sub-themes identified, with supporting quotes.

**Table 2:** Themes and example quotes from qualitative interviews

Theme	Sub-Theme & Description	Supporting Quote
Acceptability (Intervention recipients)	*Age & Tradition* Perception that older, more traditional customers (usually male) tend to prefer meat-based meals, while younger customers are more open to trying new foods ; this was reported both by older male customers themselves (particularly in relation to manual labour roles) and by other participants based on observations in the canteen.	*“it’s more the women or the healthier conscious guys over your assembly*,* you know*,* assembly workers*,* basically. It’s more the office staff that are trying different foods.”* Recipient
		*“he goes there and he has a habit of eating what he’s been eating for a long time*,* if he’s not given that*,* he’ll definitely not feel happy about it and it’ll impact his work as well”* Recipient
	*Low intervention intrusiveness* Perception that the intervention was not a significant change from standard provision, and did not pressurise customers into choosing vegetarian options	*“I think this is a very good way of encouraging some stuff without consciously putting it out there. Because some people might not always want to be told what to eat you know*,* so or a way to guide them to eat something*,* so this is an organic way*,* maybe steps like these*,* these are a good way I would say. I don’t know what more*,* but some discrete and some polite steps like these might encourage more.”* Recipient
Acceptability (Intervention implementers)	*Ease of implementation* Perception that the intervention is reasonable and straightforward to implement, as it does not significantly deviate from normal service	*“There’s always that sort of trepidation at first…is it going to create more work…but realistically*,* it just took a bit of sort of juggling around with menus and things like that. But it wasn’t that much extra work…”* Implementer
	*Additional workload* Perception that the intervention increased staff workload, as producing vegetarian meals was viewed as more labour intensive and an inefficient use of time if the meals were not purchased	*“…and it also was a time-consuming effort. You chop all these vegetables and then…no one would buy” Implementer*
	*Existing workplace food-related interventions* Perception that previous experience with healthy and sustainable food interventions facilitated the implementation of the current intervention, reducing the learning curve and increasing acceptability	“*I was making changes to the menu so it was reducing one meat item and replacing it with a vegetarian item. It was sort of easier for me to do anyway…with the* [catering provider’s healthy food menu options], *it was easy to sort of tag it onto that…”* Implementer
Barriers	*Food waste* Perception that the intervention contributed to increased food waste, as pre-prepared yet unsold meals were thrown away by staff	*“I think I was making*,* probably six to eight of it. Because we had three*,* like*,* lunch*,* lates*,* and nights. And then obviously*,* it wasn’t going so we cut it down to like four portions.”* Implementer
	*Time to eat* Feeling under time pressure during very short lunch breaks	*“*Interviewer: *So*,* would you have enough time to walk up to the cafeteria*,* buy some food*,* sit down and eat it*,* and not feel too rushed? Recipient: I’d put a question mark on that if I’m totally honest… I would say not*,* but that is*,* unfortunately*,* that’s the way the business is.” Recipient*
	*Job demands* Perception that manual labour roles require protein-rich, meat-based meals	*“I nearly went for the vegetable…but I backed off and had the chicken because I also need the protein because there’s a lot of strenuous work involved and lifting and carrying.”* Recipient
Optimisation	*Positive relationships with cafeteria staff * Perception that positive relationships with cafeteria staff increases intervention acceptability	*“if I come up and I want something and he’s only got a vegetarian option of it*,* or vegan option of it*,* just the look on his face will make me say*,* go on then…I’ll have it…He’s a good salesman*,* he’s not just the guy that serves the food.”* Recipient
	*Accessible information* Perception that the intervention would benefit from incorporating awareness and engagement strategies, particularly to highlight its goals	“*I would say probably labels and probably a bit more information would*,* I think*,* help speed up the change if that makes sense […] If we say*,* for example*,* there was 50% less CO2 emissions in this*,* so that immediately helps us get a perspective.*” Recipient
	*Pricing * Perception that pairing the intervention with both relative and actual price reductions for vegetarian meals would enhance uptake	“*I think getting people to try it*,* the price is a key point for just getting people interested.*” Recipient
	*Taste preferences* Perception that meeting the tastes of customers would increase intervention engagement	*“if they are offered something that tastes surprisingly good. It might taste better than other meals that are worse for the environment that they would normally buy.”* Recipient

## Discussion

This stepped-wedge cluster-randomised controlled trial evaluated an intervention to increase vegetarian meal sales in cafeterias by increasing their availability. During intervention weeks, the likelihood of selling a vegetarian meal increased by 41%, relative to control periods, whereas the environmental impact and nutritional value of meals sold improved overall, indicating that availability interventions in workplace cafeterias can be effective in shifting eating behaviour toward more plant-based, sustainable, and healthy diets. We found no evidence of unintended negative effects on business metrics such as the number of meals sold, revenue, and food waste.

Our three sensitivity analyses provided further evidence that the increase in vegetarian sales proportion was caused by the intervention. First, a per-protocol analysis, limited to days when implementation was confirmed by our fidelity checks, showed a stronger effect size, suggesting the intervention itself, rather than other factors, drove the observed change in vegetarian sales proportion. Second, a secular time trend analysis of vegetarian sales in matched, non-participating sites did not detect a change in line with the intervention schedule, suggesting that the effects observed in intervention sites were unlikely to be an artefact of temporal trends in vegetarian sales. Third, an extended time period analysis, including additional non-randomized periods produced a similar effect size as the primary analysis but with smaller confidence intervals, suggesting that the intervention’s impact is stable over time while it remains in place. Secondary analyses on environmental impact of meals showed an improvement across four sustainability metrics. Environmental impact estimates were based on global averages as supply chain data was not available. While relative differences between foods are consistent across contexts, absolute impacts vary within and between countries [28]; our results therefore reflect the direction and relative magnitude of change rather than absolute impacts. Previous vegetarian availability studies have often not reported environmental outcomes [21, 23, 24]. Those that have, focused exclusively on GHG emissions and found reductions between 5% [30] and 20% [22]. Our GHG emission reductions of 8.5% (given baseline emissions of 1.88 kg CO_2_-eq and effect of -0.16 kg CO_2_-eq), along with reductions in three other sustainability metrics strengthen previous findings that increasing the availability of vegetarian meals can meaningfully reduce the environmental impact of food outlets.

The intervention was associated with improvements in the nutritional profile of meals sold. Per-meal energy, total fat, saturated fat, salt, carbohydrates, and protein were all lower during intervention periods, while fibre and sugar content did not significantly change. Reductions in energy, saturated fat, and salt are consistent with UK dietary recommendations aimed at reducing cardiometabolic risk. Protein content per meal was approximately 2 g lower, reflecting concerns raised by some customers – particularly those in physically demanding roles – about the adequacy of vegetarian meals; however, this difference is small relative to typical daily protein requirements in the UK (45–55 g/day [31]). Although portion sizes were smaller during intervention periods, re-analysing outcomes per 100 g produced similar results, indicating that findings were not driven solely by portion size. Evidence on nutritional impacts of nudging and availability interventions remains limited, with most prior studies not assessing nutritional outcomes [20] and mixed findings reported where they have [22, 32]; in this context, our results provide novel evidence that increasing vegetarian availability can improve nutritional outcomes.

Cafeteria managers were free to choose which dishes to prepare within constraints on the number of meat-based and vegetarian options offered. Although plant-based ingredients are generally healthier and more sustainable than meat [33], this is not guaranteed for all substitutions, for example if a chicken curry is replaced with a cheese pizza. Giving simple instructions to intervention implementers (‘replace one meat-based with a vegetarian meal’) avoids defining what constitutes a ‘healthy’ or ‘sustainable’ meal and was sufficient to achieve nutrition and sustainability improvements here, but may not always do so (see Arrazat and colleagues [22] who reported reduced nutritional quality) and perhaps stronger effects could be achieved if more detailed instructions had been given on which meals to prepare. Future studies should compare this simple approach with interventions providing more detailed instructions on meal composition.

One surprising finding was the increased proportion of vegetarian products sold outside of the main meal range at lunch. This may suggest a spillover effect of perceived social norms of vegetarianism, implicitly communicated via the higher availability of vegetarian main meals – one suggested mechanism of action of availability interventions [18]. On the other hand, cafeteria managers may have incidentally increased availability of vegetarian foods in ranges outside of main meals. This could not be tested because we only had main meal menus available and could not ascertain if vegetarian meal availability changed in any other food ranges. However, size and confidence in this effect are very small (OR = 1.06, 95% CI = 1.00 to 1.13). Importantly, there was no suggestion of a compensation effect of increased meat purchases in non-meal lunch items due to the increased purchases of vegetarian main meals.

Interviews with intervention recipipents (customers) indicate that the intervention did not lead to significant customer frustration: customers felt that the intervention was non-intrusive, encouraging vegetarian choices without exerting pressure. Concerns about physical labour requiring meat/high-protein meals may have led some customers to keep purchasing meat meals but our analyses of sales data suggest no resultant overall loss of sales. In some cafeterias, customers reported that very short lunch breaks may have interfered with the intervention effect. Although nudging interventions require minimal cognitive effort, high time pressure can reinforce habitual food choices, leading customers to seek familiar options rather than attend to new vegetarian meals [34]. Cafeteria staff comments on ease of implementation were mixed. Overall, there seemed to be a common theme of initial worry about additional workload, food waste, and customer satisfaction, but most staff reported that the intervention required minimal deviation from standard service once it was set up, confirming feasibility. Some staff raised concerns about food waste specifically from unsold vegetarian meals – this was not confirmed by our quantitative analysis of food waste records but the large confidence intervals suggest too much variation in the data to draw final conclusions. Consistent with our findings, a university canteen study found no difference in food waste following a vegetarian meal availability intervention [22], while an observational hospital study reported lower plate waste for vegetarian than meat-based meals [35]. Food waste should therefore continue to be monitored in future availability interventions.

Our findings extend a growing body of evidence that availability interventions can reduce meat consumption in out-of-home food settings. Previous studies in university cafeterias have shown that increasing the proportion of vegetarian options can lead to meaningful reductions in meat sales without negatively affecting overall food purchases or customer satisfaction [22, 23]. Our study adds to this by demonstrating similar effects in worksite cafeterias, where the socio-economically broader customer population may have had less prior motivation or opportunity to reduce meat consumption – for example, due to time constraints or lower exposure to education or information about health and climate impacts – reflected in some sites’ very low vegetarian sales proportions at baseline. Our intervention may have been effective in these populations because the mechanisms of action of availability interventions – preference, social norms, and attention [16] – are mostly processed automatically, affecting behaviour change without requiring prior intention, resources, or additional effort from the customer. However, our qualitative analysis suggests that effectiveness of the intervention may have been lower in older male customers and those with more traditional values, warranting further investigation into the role that demographics play in availability interventions.

Availability interventions have rarely been studied in worksite settings [24, 32] and existing evidence comes largely from non-randomised field studies that altered the availability of lower-energy options across multiple food categories rather than specifically increasing vegetarian meal availability, and have yielded mixed results [24]. This study focused on vegetarian main meals (a simpler instruction to cafeteria managers), used a more thorough randomised controlled design, extended the secondary measures to include metrics of sustainability, health, and business impact, and was further supported by a qualitative process evaluation. This provides a comprehensive picture, showing that increasing vegetarian meal availability not only increases their sales but can also reduce the environmental impact and calorific value of meals sold, without adverse business outcomes, and while maintaining customer satisfaction.

This study has several strengths. It is a robust randomised trial evaluating a vegetarian meal availability intervention in settings involving a diverse range of customers. The stepped-wedge cluster design enabled strong causal inference while preserving statistical power. Use of sales data avoided self-report bias, and the inclusion of environmental, nutritional, and business outcomes allowed us to evaluate multiple dimensions of impact. Sensitivity analyses and qualitative interviews added depth and confidence. Nonetheless, the study has limitations. Anonymised sales data prevented us from analysing individual-level variation or equity of impact. Although availability interventions are generally considered more equitable than information-based approaches [36], we could not quantitatively determine whether effects differed by socio-economic group. Most sites involved relatively captive audiences, which may have enhanced exposure to the intervention and therefore limits generalisability of our findings. Finally, although we adjusted for time trends and included a matched control analysis, effect sizes from stepped-wedge designs are susceptible to residual confounding by temporal trends [37] and future studies should use a parallel-group RCT.

## Conclusions

Our findings provide clear guidance for implementing availability interventions in workplace food settings. Initial concerns among cafeteria staff about increased workload, food waste, or customer dissatisfaction did not materialise, suggesting that perceived barriers may be greater than actual ones. This may have led site managers to not respond to or decline the study invitation and limited the impact of this intervention. Sites that did take part received a high level of support through weekly phone calls, advance menu checks, and a site visit. Together, this suggests that to scale up this intervention, a top-down approach – for example, embedding vegetarian meal availability targets in catering contracts or organisational food policies – could help circumvent these concerns and ensure consistent implementation. However, such an approach must sit alongside wider food system enablers such as vegetable procurement practices and supply chain capacities. Additionally, and drawing on qualitative insights, initial uptake of vegetarian meals may be improved by pairing increased availability with price incentives and ensuring vegetarian options are perceived as tasty and satisfying. Together, these features could increase customer engagement while maintaining feasibility for staff.

Future studies testing availability interventions should focus on testing the generalisability of this intervention in less captive food environments, where customers may have easier access to alternative food options, and monitor potential loss of customers to alternative food outlets. Additionally, research in more controlled settings should test whether the effectiveness of availability interventions can be improved by manipulating meal price and taste, as suggested by our qualitative analysis. Finally, individual-level sales data, linked to socio-economic background should be collected to ascertain if the effect of availability interventions applies equitably to all intervention recipients regardless of socio-economic status, and the extent to which these are effective relative to the recipient’s history of vegetarian meal selection.

This study provides robust and holistic evidence that increasing vegetarian meal availability can shift food choices toward more sustainable and healthy options, without negative business impacts. As food systems seek scalable solutions to address climate and health challenges, availability interventions offer a low-cost, acceptable, and effective tool for real-world impact.

## Supplementary Information

Supplementary Material 1.

## Data Availability

Data for this study cannot be shared as the catering provider has not granted permission for data sharing. The full study protocol and analysis plan can be found at the Open Science Framework under DOI 10.17605/OSF.IO/DSFGZ.

## Abbreviations

GHG: greenhouse gas
CO_2_-eq: CO_2_-equivalent
GBP: Great British Pounds
RCT: Randomised Controlled Trial
PO_4_-eq: PO_4_-equivalent
L-eq: litre-equivalent
